# Autoimmune Encephalitis Induced by SARS-CoV-2 Infection Treated with Thymectomy

**DOI:** 10.1016/j.atssr.2024.04.035

**Published:** 2024-05-28

**Authors:** Cristian Puerta, Nolan M. Winicki, Casandra E. Besse, Yu Zhang, Mark Onaitis, Joshua Boys, Jessica Hudson, Patricia A. Thistlethwaite

**Affiliations:** 1 Division of Cardiothoracic Surgery, University of California, San Diego, California

## Abstract

Thymomas have been associated with the generation of paraneoplastic autoantibodies to neurogenic epitopes, collapsin-response-mediator protein-5 receptor (CRMP-5) and alpha-amino-3-hydroxyl-5methyl-4isoxazolepropionic acid receptor (AMPAR), in patients with acute viral infection. We report a patient with thymoma and myasthenia gravis, with SARS-CoV-2 infection, who became comatose secondary to autoimmune encephalitis. Plasmapheresis, high-dose steroids, pyridostigmine, eculizumab, and rituximab did not restore neurologic function. Robotic thymectomy with thymoma resection rendered CRMP-5 and AMPAR antibody titers undetectable and restored neurologic function. Thoracic surgeons should be aware of this rare condition and know that thymectomy is a potential cure for autoimmune-induced encephalitis in patients with thymoma.

Although SARS-CoV-2 infection predominantly affects the lungs, it can affect other organs by inciting acute inflammation, cytotoxic T-cell expansion, and organ-destroying autoantibody formation.^1^ A high incidence of new-onset autoimmune disorders has been seen in individuals after acute SARS-CoV-2 infection, including type 1 diabetes, myocarditis, thrombocytopenia, hemolytic anemia, and Guillain-Barré syndrome.^1^ Recent reports suggest that the SARS-CoV-2 virus can disturb self-tolerance and trigger autoimmune responses through cross-reactivity with host cells. Several pathways have been hypothesized to participate in this process, including molecular mimicry, epitope spreading, bystander activation, T-cell signaling, upregulation of cytokines and costimulatory molecules, and immortalization of infected B-cells.^1^

Patients with underlying autoimmune disease, such as myasthenia gravis, who contract SARS-CoV-2 may face synergistic impairment of their immune response.^2^ Fifteen percent of patients presenting with thymoma and myasthenia gravis present with a paraneoplastic syndrome other than myasthenia gravis.^3^ By clinical history, most of these paraneoplastic syndromes occur in the setting of or shortly after acute viral infection.

We describe the case of a patient with recently diagnosed myasthenia gravis with thymoma, in whom SARS-CoV-2 infection triggered a paraneoplastic syndrome, through the generation of autoantibodies to 2 neurogenic epitopes, collapsin-response-mediator protein-5 (CRMP-5) and alpha-amino-3-hydroxyl-5methyl-4isoxazole propionic acid receptor (AMPAR).^4^ This process induced rapid-onset encephalitis and coma. Thymectomy ameliorated anti-CRMP-5 and anti-AMPAR antibodies and restored complete neurologic function 2.5 months after operation.

A 31-year-old male individual with a known history of acetylcholine receptor-positive myasthenia gravis with thymoma (Figure 1) presented to the emergency department with a one-day history of memory loss, hallucinations, dysphagia, ataxia, involuntary movements, and tachypnea. A rapid nasal swab COVID-19 test was positive. Over the next 12 hours, he became unresponsive and required urgent intubation for airway protection. Brain computed tomography and magnetic resonance imaging with gadolinium showed no abnormalities. Serum testing showed positive antibody titers to CRMP-5 and AMPAR. Cerebrospinal fluid analysis revealed leukocytosis with a lymphocytic predominance, as well as elevated titers of CRMP-5 (1:128) and AMPAR (1:256) antibodies consistent with autoimmune encephalitis. There was an absence in the cerebrospinal fluid of other antibodies to the neurogenic and viral epitopes: *N*-methyl-d aspartate, myelin oligodendrocyte glycoprotein, amphiphysin, gamma-aminobutyric acid, glutamic acid decarboxylase-65, contactin-associated protein, SARS-CoV-2-spike protein, and SARS-CoV-2 nucleocapsid protein. The patient’s electroencephalogram demonstrated generalized slowing with focal epileptic discharges. Initially, he was treated with 3 sessions of plasmapheresis and started on pyridostigmine, eculizumab, and prednisone for presumed myasthenic crisis; however, his comatose state persisted. Rituximab was initiated, had no effect, and was discontinued after 2 weeks. Tracheostomy was performed for long-term airway control.

**Figure 1:**
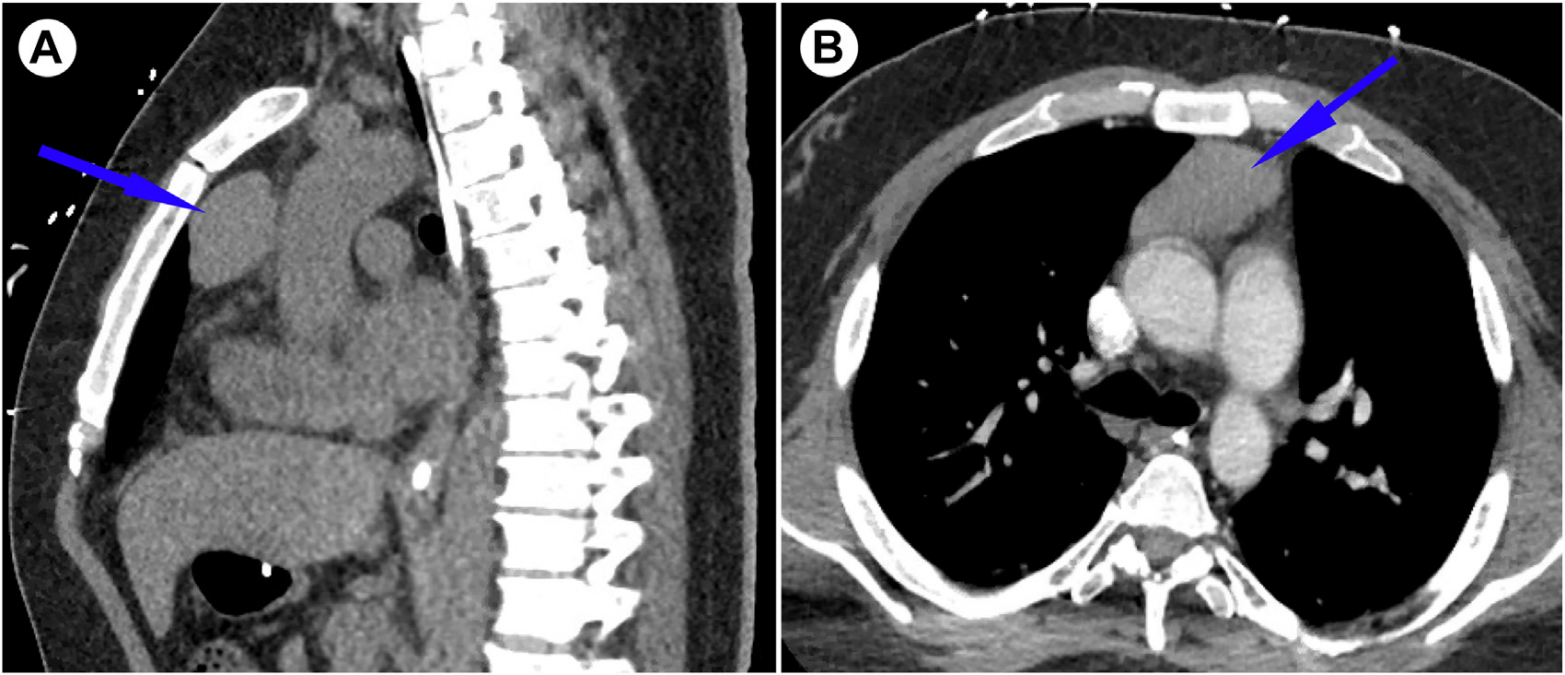
(A) Computed tomography sagittal section of the thorax shows thymoma (blue arrow) in the anterior mediastinum. (B) Transverse section of the same.

Uncomplicated robotic thymectomy with removal of thymoma was performed through the right chest. Pathologic analysis revealed Masaoka stage IIA, type B2 thymoma with negative resection margins (Figure 2). Over the next 2.5 months, CRMP and AMPAR antibody titers declined and were eventually undetectable. The patient regained consciousness with resolution of all neurologic deficits. After tracheostomy decannulation, he was discharged on a prednisone taper, with a normal neurologic exam and normal muscle strength.

**Figure 2:**
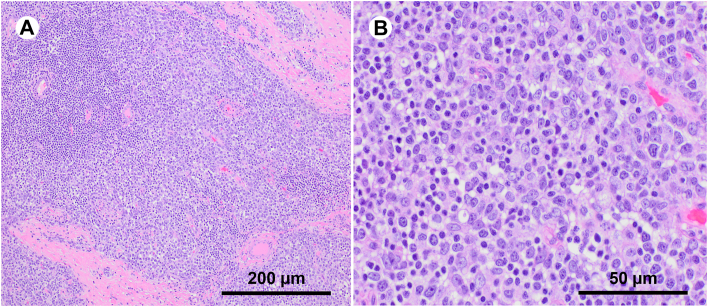
Hematoxylin and eosin staining of surgical specimen shows type B thymoma (A = 10x; scale bar = 200 μm), (B = 40x, scale bar = 50 μm).

## Comment

Surgical thymectomy for thymoma is an effective method to control autoimmune paraneoplastic encephalitis in patients with positive CRMP-5 and AMPAR antibodies in the setting of acute SARS-CoV-2 infection. Encephalitis from CRMP-5 or AMPAR autoantibodies is a rare condition with a mortality rate of more than 60%.^4^ When seen, it is in patients with malignancy who experience acute viral infection.^5^ Small cell lung cancer or thymoma is present in 80% of cases.^4^

Autoimmune paraneoplastic encephalitis is postulated to result from immune cross-reactivity between thymic epitopes and nervous system components with the formation of antibodies and antigen-specific cytotoxic and helper T-lymphocytes.^4^ Patients may present with sudden onset of cerebellar ataxia, confusion and depressed consciousness, sensorimotor neuropathy, or obtundation.^5^ Paraneoplastic encephalitis related to thymoma with CRMP-5 and AMPAR antibodies has been reported in 4 cases in the world’s literature to date^4^ and none in the setting of acute SARS-CoV-2 infection. Of the previously 4 reported cases prior to the SARS-CoV-2 pandemic, all had viral infection as a precipitating event, with afflicted patients experiencing a rapidly progressive course, with only one survivor.^4^

The mechanism of how viral infection potentiates autoimmune encephalitis associated with thymoma is not known. Cytokine- and interferon-mediated neuroinflammation in response to SARS-CoV-2 has been postulated to play an inciting role in the pathogenesis of autoimmune paraneoplastic encephalitis.^6^ Severe cases of SARS-CoV-2 infection are known to induce the overproduction of inflammatory cytokines, such as IL-2R, IL-6, IL-8, IL-10, and TNFα, as well as a type 1 interferon response with elevated levels of IL-α and IL-β.^6^ Overproduction of inflammatory cytokines and interferons has been shown to increase permeability of the blood-brain barrier, leading to viral, T-cell, antigen-presenting dendritic cell, and monocyte permeability into the cerebrospinal fluid.^7^ Autopsy studies of autoimmune paraneoplastic encephalitis in thymectomy patients have shown extensive CD8+ cytotoxic T-cell, CD4+ helper T-cell, CD20+ T-cell, CD14, and CD16 monocyte infiltration of the central nervous system with autoantibody tropism to neuronal cells.^7^

Autoantibody formation is postulated to occur by 1 or more of 3 mechanisms: molecular mimicry, epitope spreading, and bystander activiation.^1^ Molecular mimicry results from a structural similarity between a self-protein and a foreign antigen. Both antigens are bound to antigen-presenting cells and recognized by the same T-cell receptors, resulting in cross-reactivity of T-helper cells, inducing activation of B-cell-producing autoantibodies and leading to release of cytokines that recruit monocytes and macrophages that mediate tissue damage. In contrast, bystander activation occurs when self-antigens are released from damaged tissues and are taken up by antigen-presenting cells and presented to naïve autoreactive, non-antigenic-specific T-cells that become responsive. These self-antigens are not normally exposed to the immune system in the absence of an infectious trigger. B-cells recognize the self-antigens and, with T-cell help, produce autoantibodies. Finally, epitope spreading occurs when viral infection damages tissue with release of multiple, new self-antigens that are presented by antigen-presenting cells to stimulate autoreactive specific T-helper cells that recognize multiple, new self-epitopes, leading to B-cell activation. We suspect that a combination of mechanisms, eventually resulting in autoantibody formation led to the rapid progression of neurologic symptoms in this patient.

In conclusion, this case demonstrates the effectiveness of surgical thymectomy for autoimmune encephalitis with positive CRMP-5 and AMPAR antibodies, in the setting of acute SARS-CoV-2 infection. Although there is a lag time between thymectomy and recovery of neurologic function, slow recovery is not a reason to withdraw care. Patients with autoimmune encephalitis precipitated by SARS-CoV-2 infection should be screened for malignancy. Aggressive surgical treatment in the setting of SARS-CoV-2 infection is indicated for this life-threatening condition. Further investigation into the molecular mechanism of SARS-CoV-2 induction of this deadly autoimmune response is warranted.
